# Potato calcineurin B-like protein CBL4, interacting with calcineurin B-like protein-interacting protein kinase CIPK2, positively regulates plant resistance to stem canker caused by *Rhizoctonia solani*

**DOI:** 10.3389/fmicb.2022.1032900

**Published:** 2023-01-04

**Authors:** Shuai Yang, Jie Li, Jie Lu, Ling Wang, Fanxiang Min, Mei Guo, Qi Wei, Wenzhong Wang, Xuezhi Dong, Yanzhi Mao, Linshuang Hu, Xiaodan Wang

**Affiliations:** ^1^Institute of Industrial Crop, Heilongjiang Academy of Agricultural Sciences, Harbin, China; ^2^Department of Plant Pathology, College of Plant Protection, China Agricultural University, Beijing, China; ^3^Biotechnology Research Institute, Heilongjiang Academy of Agricultural Sciences, Harbin, China; ^4^Institute of Crop Cultivation and Tillage, Heilongjiang Academy of Agricultural Sciences, Harbin, China

**Keywords:** Calcineurin B-like protein, CBL-interacting protein kinases, disease resistance, expression profile, regulation, *Rhizoctonia solani*

## Abstract

**Introduction:**

Calcium sensor calcineurin B-like proteins (CBLs) and their interacting partners, CBL-interacting protein kinases (CIPKs), have emerged as a complex network in response to abiotic and biotic stress perception. However, little is known about how CBL-CIPK complexes function in potatoes.

**Methods:**

In this study, we identified the components of one potato signaling complex, StCBL4–StCIPK2, and characterized its function in defense against *Rhizoctonia solani* causing stem canker in potato.

**Results:**

Expressions of both *StCBL4* and *StCIPK2* from potato were coordinately induced upon *R. solani* infection and following exposure to the defense genes. Furthermore, transient overexpression of *StCBL4* and *StCIPK2* individually and synergistically increased the tolerance of potato plants to *R. solani* in *Nicotiana benthamiana*. Additionally, the transgenic potato has also been shown to enhance resistance significantly. In contrast, susceptibility to *R. solani* was exhibited in *N. benthamiana* following virus-induced gene silencing of *NbCBL* and *NbCIPK2*. Evidence revealed that StCBL4 could interact in yeast and *in planta* with StCIPK2. *StCBL4* and *StCIPK2* transcription was induced upon *R. solani* infection and this expression in response to the pathogen was enhanced in *StCBL4-* and *StCIPK2-*transgenic potato. Moreover, accumulated expression of pathogenesis-related (*PR*) genes and reactive oxygen species (ROS) was significantly upregulated and enhanced in both *StCBL4-* and *StCIPK2-* transgenic potato.

**Discussion:**

Accordingly, *StCBL4* and *StCIPK2* were involved in regulating the immune response to defend the potato plant against *R. solani*. Together, our data demonstrate that *StCBL4* functions in concert with *StCIPK2*, as positive regulators of immunity, contributing to combating stem canker disease in potato.

## Introduction

Plants have evolved tightly regulated signaling pathways that allow them to respond to threats they encounter during exposure to complex and varying environments (Verma et al., 2021). To cope with numerous environmental stimuli, plants rely on a series of complex signal transduction mechanisms to perceive, transduce, and respond to different stresses (Yin et al., 2017). As a secondary messenger in plants, calcium (Ca^2+^) links various signal pathways together and participates in many biochemical reactions, with altered Ca^2+^ levels observed when plants are exposed to biotic and abiotic stressors (Xu et al., 2022). Importantly, transient changes in intracellular Ca^2+^ concentration are captured by Ca^2+^ sensors that subsequently regulate signaling pathways involved in plant growth and development. Ca^2+^ sensors include two types of Ca^2+^ sensor proteins with distinct structural features and functions: sensor relay proteins and sensor responder proteins. Sensor relay proteins include calmodulin-like proteins (CMLs) and calcineurin B-like proteins (CBLs), while sensor responder proteins include calmodulins (CAMs) and calcium-dependent protein kinases (CDPKs; Tang et al., 2020). Sensor relays (e.g., CMLs and CBLs) lack kinase activity but can specifically target downstream proteins to transmit perceived calcium signals generated in response to various environmental stimuli and developmental processes. In contrast, sensor responder proteins (e.g., CaMs and CDPKs) possess kinase activity and all of the abovementioned sensor relay activities.

As a family of Ca^2+^ sensor proteins, CBLs are generally found in plants, but not in animals or fungi. CBLs contain a crucial structural component consisting of common elongation factor hand domains (EF hands). EF hands function as calcium-binding sites that capture Ca^2+^ while engaging in specific binding interactions with NAF/FISL domains present within C-terminal regions of CBL-interacting protein kinases (CIPKs). CIPKs comprise a group of plant-specific serine/threonine (Ser/Thr) kinases that belong to the SnRK3 protein family. These kinases play crucial roles in signal transduction *via* CBL–CIPK modules (Ma et al., 2020) to thereby control affinities and activities of numerous ion transporters. In contrast, the N-terminal MGCXXS/T motif in some CBL proteins mediates lipid modification *via* myristoylation and palmitoylation that direct CBL-CIPK complex migration to precise cellular membrane target regions where they become anchored to the membrane (Weinl and Kudla, 2009). *Arabidopsis* CBLs include CBL1, 4, 5, and 9, which localize to the plasma membrane (Batistic et al., 2010).

Calcineurin B-like protein–interacting protein kinase complexes of many plant species, including model plants, crop plants, and several horticultural plants, have been extensively studiedconcerning their regulation of diverse responses to abiotic stresses (Dong et al., 2021). One such complex, which was first identified in *Arabidopsis thaliana*, was initially found to participate in the Salt Overly Sensitive (SOS) signaling pathway, whereby CBL4 (SOS3) was shown to interact and form a complex with CIPK24 (SOS2). Once formed, the CBL4-CIPK24 complex was observed to migrate to the plasma membrane, where it activated the Na^+^/H^+^ antiporter (SOS1) located on the plasma membrane and the vacuolar H^+^-ATPase, resulting in enhanced salt tolerance (Ishitani et al., 2000). Subsequently, other complexes were discovered in *Arabidopsis* (CBL1/CBL9-CIPK23) that were observed to localize to the plasma membrane and regulate K^+^ in roots and stomatal guard cells by modulating K^+^ channel Arabidopsis K^+^ Transporter 1 (AKT1) activity (Cheong et al., 2007). Furthermore, overexpression of OsCBL8 and OsCIPK15 in rice was found to enhance salt tolerance (Xiang et al., 2007). Conversely, expression of OsCIPK31 in tobacco plants was found to participate in the generation of diverse signals in response to cold, salt, light, cytokinins, and sugars (Kim et al., 2003). More recently, TaCIPK23 has been shown to participate in wheat abscisic acid (ABA) and drought stress responses by mediating crosstalk between ABA-induced signaling and other pathways associated with adaptation to drought (Cui et al., 2018). In potato, StCIPK10 has been shown to enhance both cellular scavenging of ROS and cellular modulation of corresponding osmoregulatory substances to strengthen plant drought and osmotic stress tolerance (Ma et al., 2021). In addition, differential expression of maize *ZmCBL4*, pea *PsCBL*, Chinese cabbage *BrCBL*, and grapevine *VvCBL* have been reported to occur after exposure of plants to various abiotic stresses (Wang et al., 2007; Jung et al., 2017; Xi et al., 2017).

Compared with studies of abiotic stress responses, studies of biotic stress responses have rarely been reported, although a few studies can be found in the literature. One such study using cultured cells of rice plants demonstrated that an interaction between OsCIPK14/15 and OsCBL4 played a crucial role in the microbe-associated molecular pattern-induced defense signaling pathway (Kurusu et al., 2010). CBL10 and CIPK6 were found to promote ROS generation as part of the tomato immune defense response triggered by an interaction between *Pseudomonas syringae* pv tomato DC3000 and *Nicotiana benthamiana* (De la Torre et al., 2013). OsCIPK30 was shown to play a critical role in enhancing rice tolerance to rice stripe virus (Liu et al., 2017), while the TaCBL4–TaCIPK5 complex was observed to positively modulate wheat resistance to stripe rust fungus through a ROS-dependent mechanism (Liu et al., 2018). Moreover, the knockdown of CaCIPK1 expression increased the susceptibility of pepper to *Phytophthora capsici*, reduced root activity, and altered the expression of defense-related genes (Ma et al., 2019). Taken together, the abovementioned results obtained from the few research studies reported to date suggest that CBL-CIPK complexes participate in biotic stress responses.

*Rhizoctonia solani* (teleomorph: *Thanatephorus cucumeris*) is one of the most dominant soil-borne necrotrophic fungal pathogens, due to its broad host range and the lack of crop resistance to this pathogen (Foley et al., 2013). *Rhizoctonia solani* is complex and divided into 14 anastomosis groups (AG1–AG13 and AGBI; Yang et al., 2017). AG3, the most destructive group, causes *Rhizoctonia* disease on potato (*Solanum tuberosum* L.; Woodhall et al., 2013), the fourth most cultivated food crop globally. In recent years, black scurf and stem canker have emerged to become the most severe soil-borne diseases, as evidenced by increasingly reduced crop yields and quality due to these diseases each year. However, efforts to combat these diseases have been hindered by a lack of knowledge about the necrotrophic pathogen infection process and the mechanisms by which plants prevent disease-induced cell death (Yang et al., 2017). In our previous study, transcriptome analysis was performed *via* RNA-Seq to investigate the potato response to *R. solani* infection, leading to the identification of two key genes, which encoded CBL4 and CIPK2 that exhibited up-regulated expression during infection (Yang et al., 2018). In this study, complete DNA sequences of the genes encoding these two proteins were cloned then their expression patterns were characterized in potato. After that, these genes were overexpressed in potato and the heterologous host *N. benthamiana* to determine whether increased expression of these genes could increase plant resistance to *Rhizoctonia* disease. *StCBL4* and *StCIPK2* were involved in regulating the immune response to defend the potato plant against *R. solani*. Therefore, the functional research of *StCBL4* in concert with *StCIPK2*, as both are positive regulators of immunity, will contribute to combating stem canker disease in potato.

## Materials and methods

### Bacterial strains, plant materials, and growth conditions

The virulent *R. solani* strain (anastomosis group: AG-3PT) used in this study was obtained from Heilongjiang Academy of Agricultural Sciences (HAAS) and cultured in potato dextrose agar (potato 200 g, Dextrose 20 g, agar 17 g, and water 1 L). Cultures were maintained on PDA medium for 1 week at 25°C, and then 5-mm-diameter mycelium plugs were obtained for inoculation of plants. In addition, *Agrobacterium tumefaciens* GV3101 was cultured in LB medium (10 g tryptone, 10 g NaCl, and 5 g yeast extract per liter).

Various potato germplasm resources studied here were grown in a climate-controlled chamber under 16 h light/8 h dark conditions at 23°C. The list of plant varieties is presented in Supplementary Table 1. In addition, *N. benthamiana* plants were grown in a climate-controlled chamber under 14 h light/10 h dark conditions and kept at 25°C and 20°C during light and dark cycles, respectively. All plants were grown on separate trays for subsequent RNA extraction, inoculation, and RT-PCR expression analysis. All experiments involving both control and treated plants were carried out using three independent biological replicate samples.

### RNA extraction, gene cloning, and sequence analysis of *StCBL4* and *StCIPK2*

Total RNA was extracted from potato leaves using TRIzol™ Reagent (Invitrogen Co., United Kingdom) according to the manufacturer’s instructions. RNA concentrations were measured using a Thermo NanoDrop 1000 spectrophotometer system (Thermo Fisher Scientific Co., United States). cDNA was synthesized using the GoScript™ Reverse Transcription System (Promega Co., United States), and then was used as a template for subsequent PCR amplifications. Based on transcript sequences, cDNA sequences encoding ORFs of *StCBL4* and *StCIPK2* were amplified with primers designed using CE Design V1.04 (Supplementary Table 2) according to amino acid sequence alignments conducted using SMART.^1^ Next, multiple sequence alignments were performed using DNAMAN 8.0 software (Lynnon Biosoft Co., United States). Next, a phylogenetic tree was constructed based on alignments of amino acid sequences *via* the neighbor-joining (NJ) method using MEGA 5.0 software. Finally, bootstrap analysis with 1,000 replicates was performed to assess the statistical reliability of each branch of the tree.

### Vector construction, subcellular localization, and potato transformation

For transient expression, complete coding sequences (CDSs) of *StCBL4* and *StCIPK2* were cloned into the pCAMBIA1300-35S-HA-RBS vector to generate constructs designated *35S:HA-StCBL4* and *35S:HA-StCIPK2*, respectively. The recombinant constructs were next introduced into *A. tumefaciens* strain GV3101, which was then used to transform *N. benthamiana* plants *via* the *Agrobacterium*-mediated transformation method, with the transformation of empty vector (EV) *35S:HA* serving as a control. To generate constructs for the luciferase complementation assay (LCA), the StCBL4-coding sequence was cloned into the pCAMBIA1300-35S-Cluc-RBS vector, and the StCIPK2-coding sequence was cloned into the pCAMBIA1300-35S-HA-Nluc-RBS vector. To construct the bait vector for the yeast two-hybrid assay, CDSs of StCBL4 and StCIPK2 were cloned into pGBKT7 (DNA-binding domain, BD) and pGADT7 (activation domain, AD) vectors, respectively. Meanwhile, pBWA(V)KS-StCBL4 and pBWA(V)KS-StCIPK2 constructs were generated and transformed into *A. tumefaciens* strain EHA105 using *Agrobacterium*-mediated transformation protocols for potato obtained from Wuhan Biorun Bio-Tech Co. Ltd. (Wuhan, China). Regenerated transgenic plants were transplanted in a standard MS medium (Murashige and Skoog, 1962) containing 50 mg/L kanamycin (Sangon Biotech Co., China). The primers used in this study are listed in Supporting Information Supplementary Table 2.

The images of subcellular localization of GFP- and RFP-tagged proteins were visualized through a Leica TCS SP8 confocal microscope (Leica Co., Germany) in *N. benthamiana* leaves expressing transiently p35S-CBL4-GFP and p35S-CIPK2-RFP constructs. Fluorescence signals were detected at 48 h after injection. GFP and RFP fluorescence was excited using argon lasers at 488 and 545 nm, and the emissions were collected within 495–545 and 580–620 nm, respectively. Relevant fluorescence data analysis and subsequent image processing of the indicated profile were conducted using Leica Application Suite X (LAS-X) software.

### Yeast two-hybrid assay

For interaction analyses of StCBL4–StCIPK2, MatchMaker yeast two-hybrid assays were performed (Coolaber Co., China). After vector construction, paired pGADT7 (activation domain, AD) and pGBKT7 (DNA-binding domain, BD) plasmids were co-transformed into yeast strain AH109 *via* the lithium acetate method according to the Yeast Protocols Handbook. The positive control was the diploid hybrid yeast containing pGBKT7-53 and pGADT7-T. Yeast colonies were grown at 30°C for 2–4 days on selection medium before being photographed. After yeast cell numbers/ml were initially quantified using a hemocytometer, they were collected and diluted to 10^2^ and 10^1^ cells/ml in sterile ultrapure water for serial dilutions.

### Luciferase complementation assay

The luciferase complementation assay was performed as described previously (Zhou et al., 2018) after Cluc and Nluc constructs were introduced into *N. benthamiana* leaves *via* the *Agrobacterium*-mediated transient expression-based method followed by co-expression of cloned genes in leaf cells for 2 days. Next, leaf disks in wells of a 96-well plate were incubated with 1 mM luciferin (Biovision Co., United States) then luminescence was measured using a microplate luminometer (Perkin Elmer Co., United States).

### Co-immunoprecipitation assay

Two days after *Agrobacterium*-mediated transient injection of p35S-StCIPK2-HA constructs into *N. benthamiana*, leaves were collected for Co-IP experiments. Total proteins were extracted from agroinfiltrated leaves using protein immunoprecipitation (IP) buffer, which contains 50 mM HEPES (pH 7.5), 150 mM KCl, 5 mM EDTA, 0.5% Triton X-100, 1 mM DTT, and a proteinase inhibitor cocktail. Samples were centrifuged and the supernatant was transferred to another 1.5 ml centrifuge tube, and about 45 μl was taken for input analysis. The remaining extracts were then incubated with 10 μl agarose-conjugated anti-GFP antibody (Lablead Co., China) for 3 h at 4°C with shaking, followed by washing beads four times with IP buffer. Finally, about 50 μl elution buffer was added for output analysis. Immunoprecipitated proteins were separated *via* SDS-PAGE then proteins of interest were detected on immunoblots *via* probing membranes with anti-HA and anti-GFP antibodies.

### *Rhizoctonia* infection assay

The *R. solani* AG-3 strain was used for plant infection and was cultured on a PDA medium at 25°C for 3 days. 5-mm-diameter mycelium plugs were incubated onto the abaxial side of detached leaves of intact *N. benthamiana* plants stored on moist tissue in sealed boxes for 24–48 h. Then inoculated leaves were photographed under UV light, and lesion areas were measured using ImageJ^2^ software. For *S. tuberosum* experiments, mycelium inoculations were performed using *in vitro* cultured plantlets, as described previously (Yang et al., 2017).

### Virus-induced gene silencing

To understand orthologous gene functions in *N. benthamiana*, phylogenetic relationships among CBLs and CIPKs from *S. tuberosum*, *N. benthamiana*, and *A. thaliana* were analyzed. Virus-induced gene silencing (VIGS) constructs were generated by cloning 300-bp PCR fragments of orthologous genes from *N. benthamiana* cDNA into the pBinary tobacco rattle virus (TRV) vector (Liu et al., 2002) between the *EcoR*I and *Kpn*I sites in the antisense orientation. A TRV construct expressing GFP, as described previously (McLellan et al., 2013), served as the negative control and a TRV construct expressing a cloned fragment of *N. benthamiana* phytoene desaturase (*NbPDS*) served as the positive control. Primer sequences are shown in Supplementary Table 2. Plants were either used in assays or to check gene silencing levels *via* quantitative RT-PCR 2–3 weeks later.

### Oxidative burst measurement

Potato plants overexpressing the abovementioned genes were grown in a climate-controlled chamber under 16 h light/8 h dark conditions at 23°C. After 3–4 weeks of growth, detached leaves were assayed for H_2_O_2_ production. H_2_O_2_ was detected *via* staining of inoculated and uninoculated leaves with 1 mg/ml 3,3′-diaminobenzidine (DAB) for 8 h in the dark, and then the leaves were destained with ethanol prior to observation (Lu et al., 2021).

### Quantitative RT-PCR analysis

Total RNA was isolated from *N. benthamiana* as described above. For *S. tuberosum* samples, RNA isolation was conducted after plants were inoculated with *R. solani* at 0, 6, 12, 24, 48, and 96 hpi (cv. Youjin-885) or 3 dpi (for different potato germplasm resources). Total RNA (5 μg) was reverse-transcribed using the GoScript™ Reverse Transcription System to generate a cDNA template. Next, cDNA was diluted tenfold for subsequent qRT-PCR analysis, using an ABI7500 Real-Time PCR System (Applied Biosystems, Carlsbad, CA) and TransStart Top Green qPCR SuperMix (TransGen, Beijing, China). Relative gene expression levels were calculated by normalization against internal controls *NbActin* (AY179605) and *StActin* (DQ252512) for *N. benthamiana* and *S. tuberosum*, respectively. All qRT-PCR experiments were performed at least three times. Relative quantification of expression levels was achieved using the 2^-ΔΔCt^ method.

### Statistical analysis

Data were obtained from three replicates and analyzed by unpaired two-tailed Student’s *t*-test with GraphPad Prism software (GraphPad Software, Inc., La Jolla, CA, United States). Values with *p* < 0.05 were considered statistically significant.

## Results

### *StCBL4* and *StCIPK2* are rapidly induced following infection of potato with *Rhizoctonia solani*

**Figure 1:**
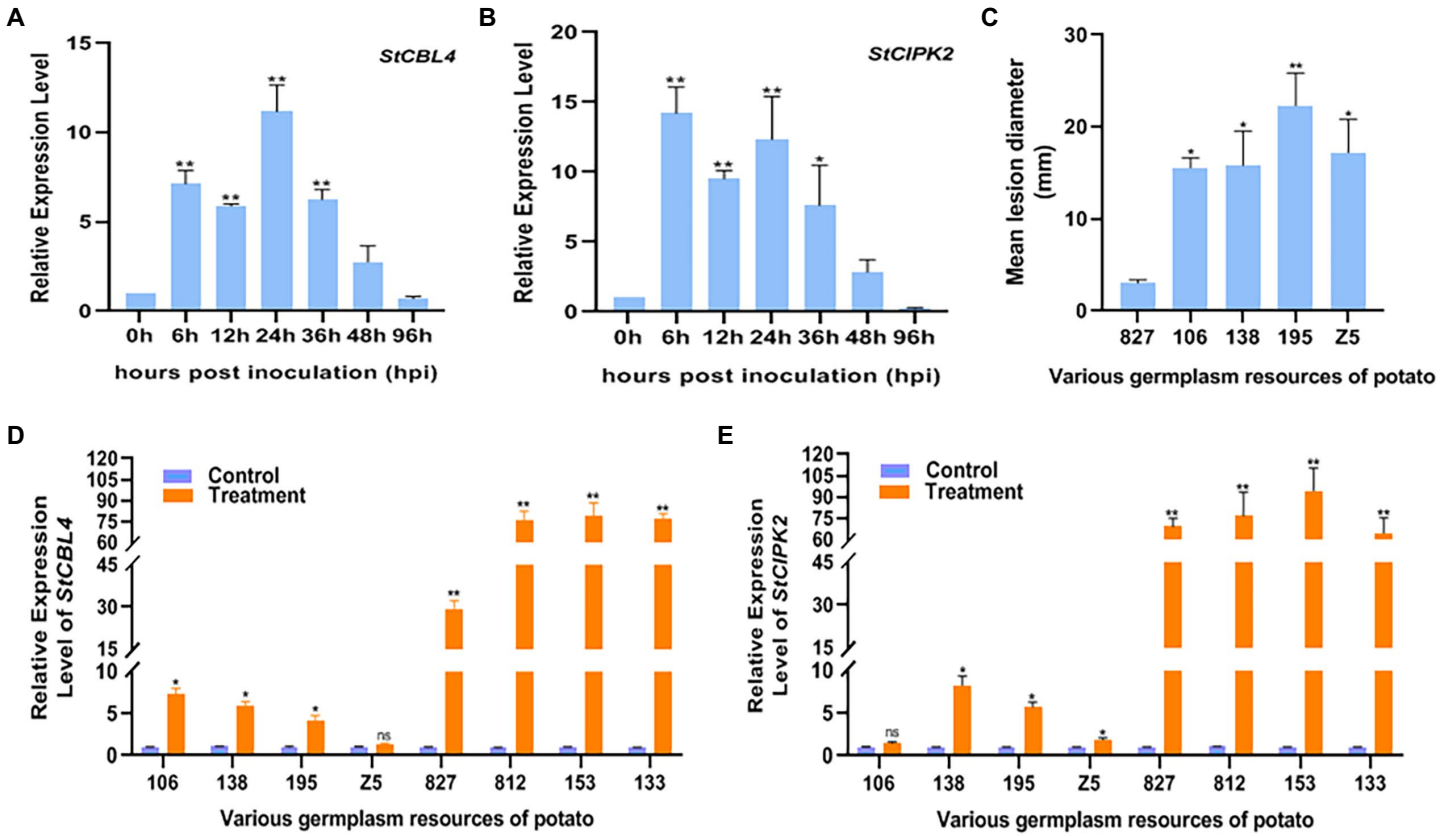
Expression profile of *StCBL4* and *StCIPK2* genes infected by *Rhizoctonia solani* in plantlet. **(A)** The relative expression level of the *StCBL4* gene at different hours post-inoculation. **(B)** The relative expression level of the *StCIPK2* gene at different hours post-inoculation. **(C)** Mean lesion diameter measured on various germplasm resources of potato after *R. solani* inoculation at 7 days post-inoculation. **(D)** The relative expression level of the *StCBL4* gene in treatment plantlets compared with control plantlets at 7 days post-inoculation. **(E)** The relative expression level of the *StCIPK2* gene in treatment plantlets compared with control plantlets at 7 days post-inoculation. The assay was performed by real-time RT-PCR. The relative mRNA levels were calculated with respect to the expression level of the WT plantlets without pathogen infection. All the experiments were repeated three times, and similar results were obtained. The data represent the means ± SD of triplicate measurements. Asterisks above the columns represent significance based on an unpaired, two-tailed Student’s *t*-test relative to the control. *p < 0.05; **p < 0.01.

In previous transcriptome analysis, preliminary screening of *StCBL4* and *StCIPK2* expression was conducted to determine whether infection of potato with *R. solani* could induce expression of these two genes. Then, relative levels of *StCBL4* and *StCIPK2* gene transcripts in cv. Youjin-885 plants were measured at 0, 6, 12, 24, 36, 48, and 96 hpi. The results revealed that the expression of *StCBL4* and *StCIPK2* was induced and upregulated significantly during the infection of *R. solani*. More specifically, the expression level of *StCBL4* was up-regulated and peaked at 24 hpi, 11.18-fold higher than the control level (Figure 1A). *StCIPK2* reached a peak level of expression 14.2-fold higher than the control level as early as 6 hpi (Figure 1B). Meanwhile, the lesion symptoms and relative levels of *StCBL4* and *StCIPK2* gene transcripts in various potato germplasm resources (with different levels of resistance to *R. solani*) at 7 dpi were measured. As shown in Figure 1C, 5 potato germplasm resources displayed the disease except the varieties of 133, 153, and 812. Compared with the mild symptom of cv. 827, the other four potato germplasm resources exhibited no less than a 5-fold disease level on stem of potato plantlet. Moreover, real-time PCR results revealed that expression levels of both *StCBL4* and *StCIPK2* were significantly up-regulated in asymptomatic potato plantlets compared with symptomatic plantlets. The latter exhibited no significant change in the expression of these genes except for *StCBL4* in potato *cv.* Z5 and *StCIPK2* in potato *cv.*106 (Figures 1D,E). Based on these observations, we speculated that *StCBL4* and *StCIPK2* might be involved in the host response to *R. solani* infection.

### Sequence analysis of *StCBL4* and *StCIPK2*

Based on transcript sequences identified *via* RNA-seq, ORF sequences of *StCBL4* and *StCIPK2* were cloned and deposited into the GenBank database under accession numbers ON383960 and ON324205, respectively. The ORF of the *StCBL4* gene is 663 bp in length and contains 4 EF-hand conserved domains and an FPSF motif that interacts with CIPKs (Supplementary Figure 1A). The predicted StCBL4 ORF contains 220 aa, a polypeptide molecular mass of 25.32 kDa, and an isoelectric point (pI) of 4.77. Results of the phylogenetic analysis indicated that *StCBL4* is highly homologous to CBL4 from *Capsicum annuum* (Supplementary Figure 1B)*. StCIPK2* is 1,413 bp in length and contains a NAF/FISL motif and a PPI motif (Supplementary Figure 2A). The predicted StCIPK2 ORF contains 470 aa, a polypeptide molecular mass of 53.01 kDa, and an isoelectric point (pI) of 8.66. Results obtained through homology analysis indicated that *StCIPK2* has high homology to *CIPK* of *Hordeum vulgare* (Supplementary Figure 2B).

### Transient expression of *StCBL4* and *StCIPK2* enhances resistance to *Rhizoctonia solani* in *Nicotiana benthamiana*

**Figure 2:**
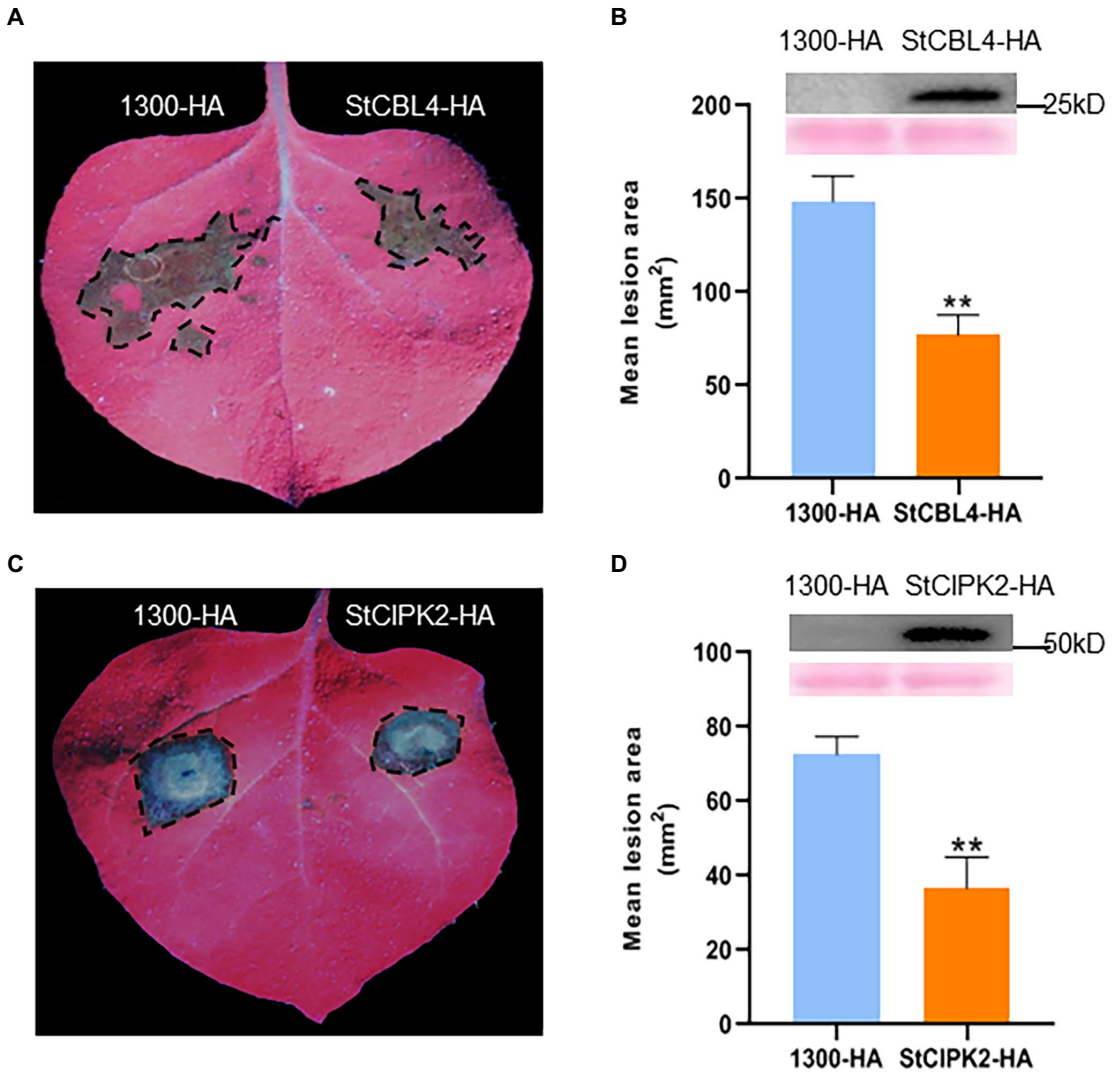
Transient expression of StCBL4 and StCIPK2 enhances the resistance to *Rhizoctonia solani* in *Nicotiana benthamiana*. StCBL4-HA, StCIPK2-HA, and 1,300-HA were expressed in *N. benthamiana* by *Agrobacterium*-mediated transient expression for 1 day. The leaves were inoculated by *R. solani* AG-3 and photographed under UV light 36 h later. **(A,C)** Representative leaf images (under UV light) by *StCBL4* and *StCIPK2* overexpression showing the extent of *R. solani* colonization on *N. benthamiana* leaves expressing each construct as indicated. **(B,D)** Immunoblotting showed the expression of StCBL4-HA and StCIPK2-HA. The lesion areas were measured and their sizes were calculated. The data represent the means ± SD of measurements (*n* > 8). The asterisks above the columns represent significance based on an unpaired, two-tailed Student’s *t*-test relative to the EV. *p < 0.05, **p < 0.01.

To investigate whether *StCBL4* and *StCIPK2* enhanced host resistance against *R. solani* infection, we transiently expressed StCBL4-HA and StCIPK2-HA in *N. benthamiana* plants using *Agrobacterium*-mediated transient expression, with pCambia1300-35S-HA empty vector (EV) as control. The results showed that StCBL4-HA and StCIPK2-HA exhibited remarkably smaller lesion areas than the EV control (Figures 2A,C), and was about 51 and 50% lower than corresponding levels in control, respectively (Figures 2B,D). These findings indicated that *StCBL4* and *StCIPK2* could positively enhance *N. benthamiana* resistance to *R. solani* infection. Furthermore, western blotting elucidated the stability of StCBL4-HA and StCIPK2-HA expressed in *N. benthamiana* (Figures 2B,D).

### VIGS of *NbCBL* and *NbCIPK2* significantly enhance plant susceptibility to *Rhizoctonia solani*

**Figure 3:**
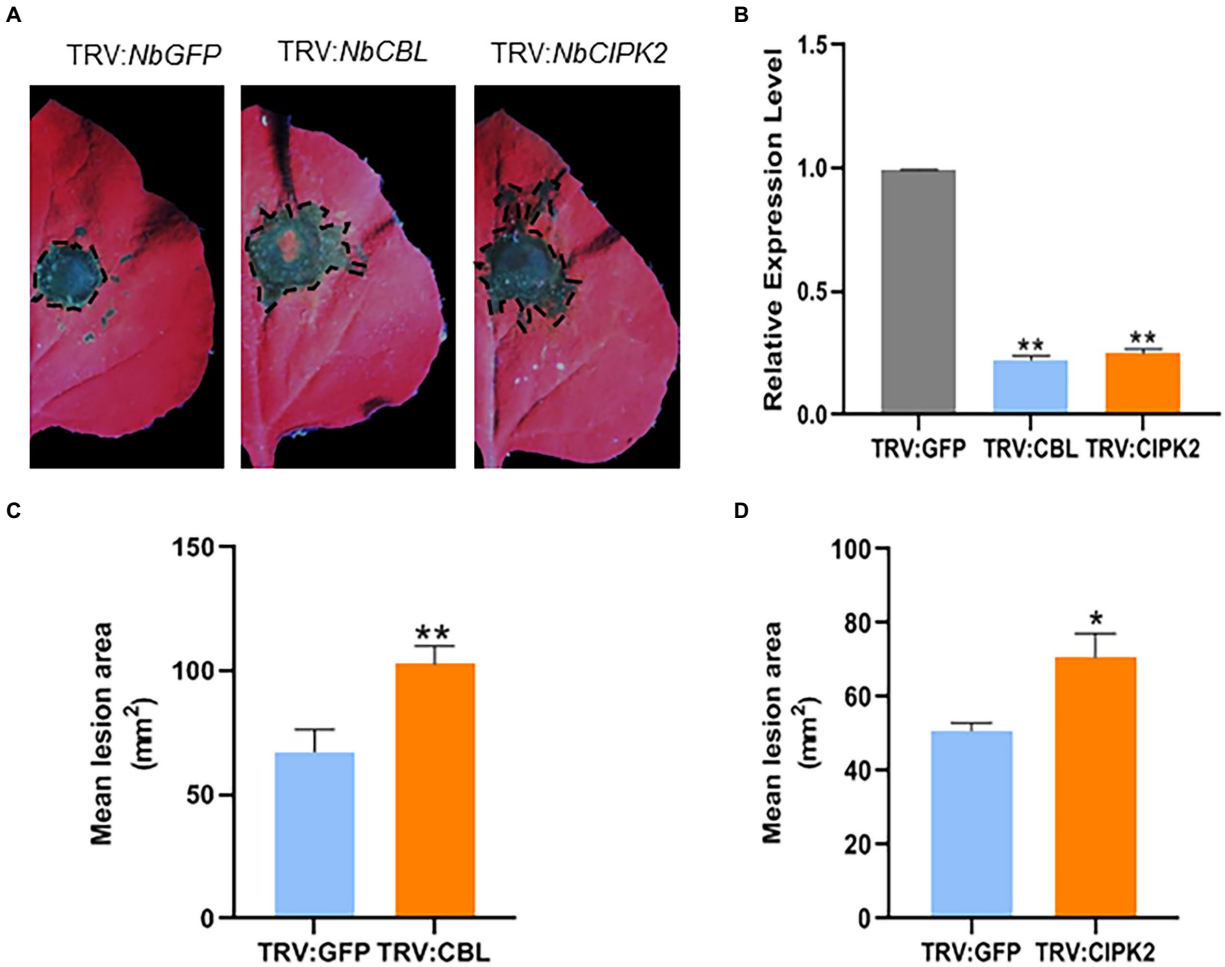
Virus-induced gene silencing (VIGS) of *NbCBL* and *NbCIPK2* significantly promotes *R. solani* colonization in *N. benthamiana*. **(A)** Representative leaf images (under UV light) showing the extent of *R. solani* colonization on *N. benthamiana* leaves expressing each construct as indicated. **(B)** TRV:NbCBL and TRV:NbCIPK2 constructs showed high silencing efficiency in *N. benthamiana* comparing with the TRV:GFP control vector. **(C,D)** Mean lesion area measured 60 h after *R. solani* inoculation. Graph showing that silencing of TRV:NbCBL and TRV:NbCIPK2 constructs significantly increases *R. solani* lesion diameter compared with TRV:GFP control vector. The data represent the means ± SD of measurements (*n* > 20). The asterisks above the columns represent significance based on an unpaired, two-tailed Student’s *t*-test relative to the V-GFP. *p < 0.05, **p < 0.01.

In order to analyze the functions of *StCBL4* and *StCIPK2*, VIGS was conducted to silence the expression of orthologous genes *NbCBL* and *NbCIPK2* which was designed using the SGN VIGS Tool^3^ in *N. benthamiana* (Supplementary Figures 3A,B, 4A,B). *NbPDS* VIGS plants began to show photobleaching after 10 days. Efficiencies of *NbCBL* and *NbCIPK2* silencing were confirmed by qRT-PCR of mRNA obtained from leaves that emerged 3 weeks after infiltration of plants with *Agrobacterium* transformed with TRV2:*NbCBL* and TRV2:*NbCIPK2* constructs. Results of qRT-PCR analysis confirmed that *NbCBL* and *NbCIPK2* were well silenced in VIGS plant leaves, with average expression levels in silenced plants found to be about 22 and 25%, respectively, lower than corresponding levels in negative control plants (TRV2:*GFP*; Figure 3B). Leaves of *NbCBL* and *NbCIPK2* VIGS plants exhibited visible disease symptoms at *R. solani* inoculation sites for 2 days. Lesion areas were measured at 60 hpi (Figure 3A). The results showed that *NbCBL* and *NbCIPK2* VIGS plants significantly increased disease lesions compared with GFP VIGS control plants (Figures 3C,D). Taken together, these results indicate that VIGS of *NbCBL* and *NbCIPK2* significantly enhance plant susceptibility to *R. solani*.

### StCBL4 interacts with StCIPK2 *in vivo*

**Figure 4:**
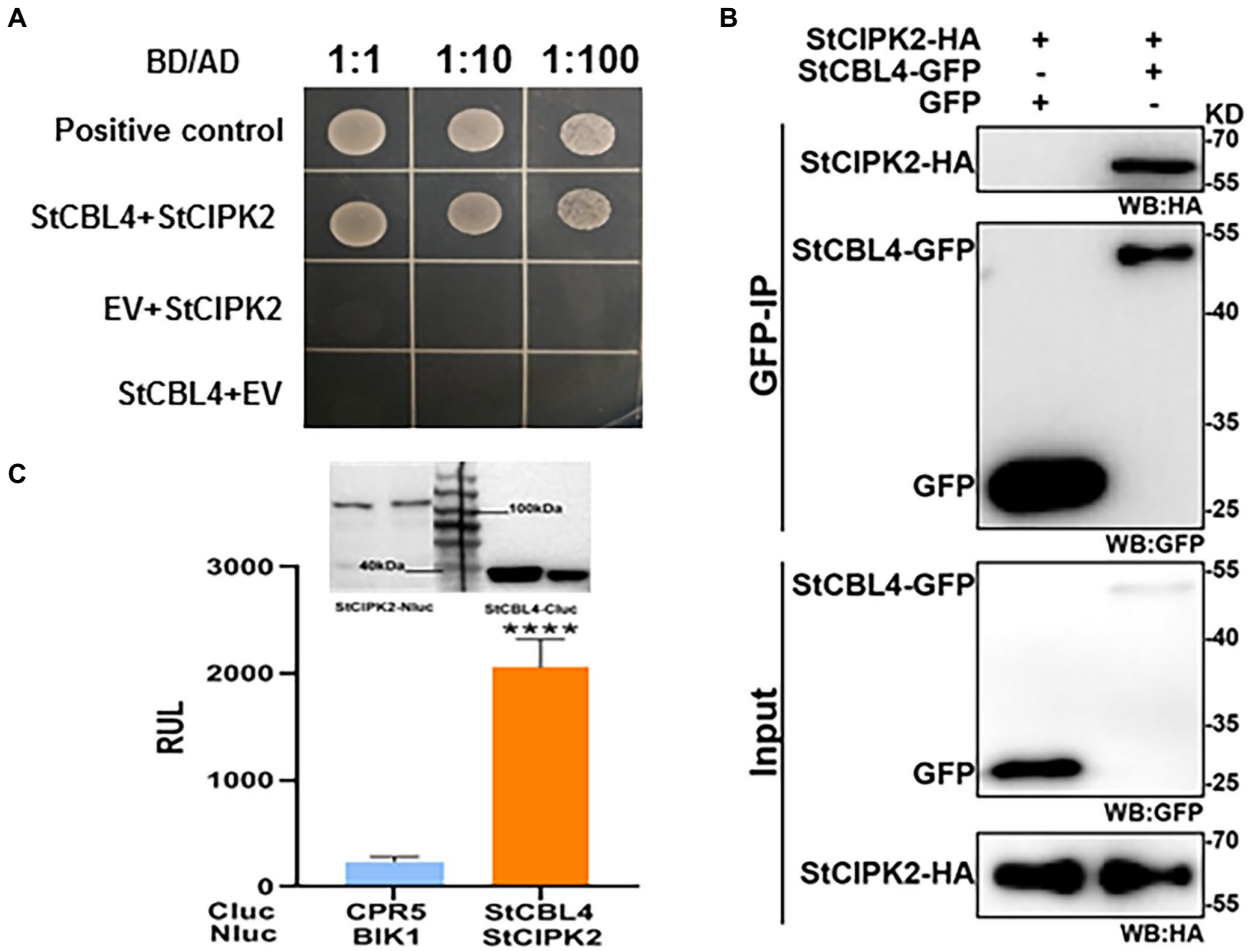
Validation of the protein interaction between StCBL4 and StCIPK2. **(A)** Yeast two-hybrid analysis of interactions between StCBL4 and StCIPK2. Yeast transformants co-expressing different bait and prey constructs were assayed on SD-Leu-Trp-His-Ade selective medium. BD-53 and AD-T were also co-transformed to serve as a positive control. **(B)** Co-IP assays were performed using an agarose-conjugated anti-GFP antibody. Input and bound proteins were detected *via* immunoblot using α-HA and α-GFP antibodies, respectively. **(C)** StCBL4 interacts with StCIPK2 by luciferase complementation assay and western blot. StCBL4-Cluc and StCIPK2-HA-Nluc constructs were transiently expressed in *N. benthamiana* plants, and the relative luminescence unit (RLU) was measured 2 days later. Cluc-CPR5 and BAK1-HA-Nluc were used as negative control (Liang et al., 2016). Immunoblotting showed the expression of StCBL4-Cluc and StCIPK2-HA-Nluc. The data represent the means ± SD of measurements (*n* > 20). The asterisks above the columns represent significance based on an unpaired, two-tailed Student’s *t*-test relative to the V-GFP. ****p < 0.0001.

Yeast two-hybrid assays were performed to investigate the proposed interaction between identified proteins. Yeast growth on a minimal selective medium lacking histidine, leucine, tryptophan, and adenine (-His/−Leu/−Trp/−Ade) indicated a physical interaction between StCBL4 and StCIPK2 (Figure 4A). In addition, Co-IP assay and luciferase complementation assay in *N. benthamiana* by *Arobacterium*-mediated transient expression was conducted to validate the interaction further. Co-IP results confirmed the Y2H results using GFP beads with anti-HA antibody, StCIPK2-HA protein was successfuliy detected in output samples while GFP was used as a negative control (Figure 4B). In addition, the split luciferase complementation assay showed that StCBL4 interacted strongly with StCIPK2 and the immunoblot analysis showed that all proteins were normally expressed (Figure 4C). All things considered, these results demonstrated that StCBL4 and StCIPK2 interact *in vivo*.

### Co-expression of *StCBL4* and *StCIPK2* significantly enhances *Rhizoctonia solani* resistance in *Nicotiana benthamiana*

**Figure 5:**
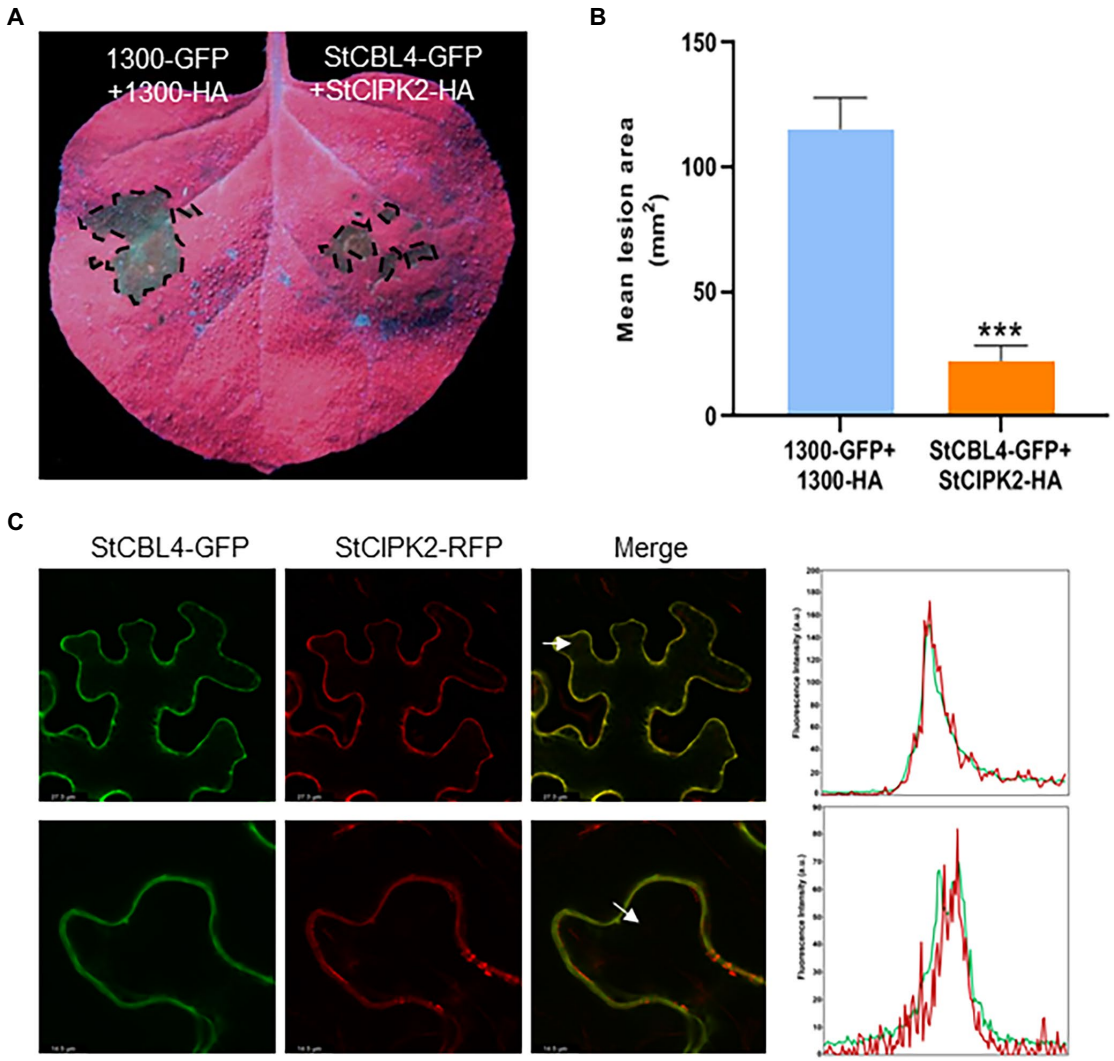
Analysis of transient co-expression and colocalization of StCBL4 and StCIPK2. **(A)** Transient co-expression of StCBL4 and StCIPK2 enhances the resistance to *R. solani* in *N. benthamiana*. Representative leaf images (under UV light) by *StCBL4* and *StCIPK2* co-overexpression showing the extent of *R. solani* colonization on *N. benthamiana* leaves expressing each construct as indicated. **(B)** The lesion areas were measured and their sizes were calculated by ImageJ software. The data represent the means±SD of measurements (*n* > 8). The asterisks above the columns represent significance based on an unpaired, two-tailed Student’s t-test relative to the control. *p < 0.05. **(C)** Confocal laser scanning microscopy showing subcellular co-localization of StCBL4-GFP and StCIPK2-RFP fusion proteins expressed transiently in *N. benthamiana* leaves. The bottom row is the magnification of the top row. White arrowheads indicate the location of a profile across the membranes and the direction of the fluorescence intensity plot of the profile (right). *y* axis: Fluorescence intensity. *x* axis: Distance. Green line, GFP fluorescence. Red line, RFP fluorescence. ***p < 0.001.

To investigate the relationship between StCBL4 and StCIPK2 to *R. solani* resistance, StCBL4-GFP were co-expressed transiently with StCIPK2-HA in *N. benthamiana* for 24 h, then inoculated *R. solani* for 36 h. pCAMBIA1300-HA and pCAMBIA1300-GFP co-expression was performed as a control, and the lesion areas of leaves were photographed at 36 hpi by UV light. As shown results in Figure 5A, leaf lesion areas of co-expressing StCBL4-GFP and StCIPK2-HA exhibited significantly smaller and lower (about 20%) than corresponding levels of the control (Figure 5B). These results revealed a higher difference compared to the single-expression of two genes, and indicated that *StCBL4* plays a positive role in the function of StCIPK2 on *R. solani* resistance.

Meanwhile, to test the subcellular localization of StCBL4 and StCIPK2, we fused a GFP tag to the C-terminus of StCBL4 and an RFP tag to the C-terminus of StCIPK2, respectively, and viewed the Agrobacterium-mediated expression in *N. benthamiana* using confocal microscopy. Confocal images showed that the co-expression of StCBL4-GFP and StCIPK2-RFP displayed colocalization in the plasma membrane. Furthermore, the related intensity plots presented coincidence peaks between GFP and RFP fluorescence, indicating the co-localization of StCBL4 and StCIPK2 (Figure 5C).

### Transgenic potato expressing *StCBL4* and *StCIPK2* enhances resistance to *Rhizoctonia solani*

**Figure 6:**
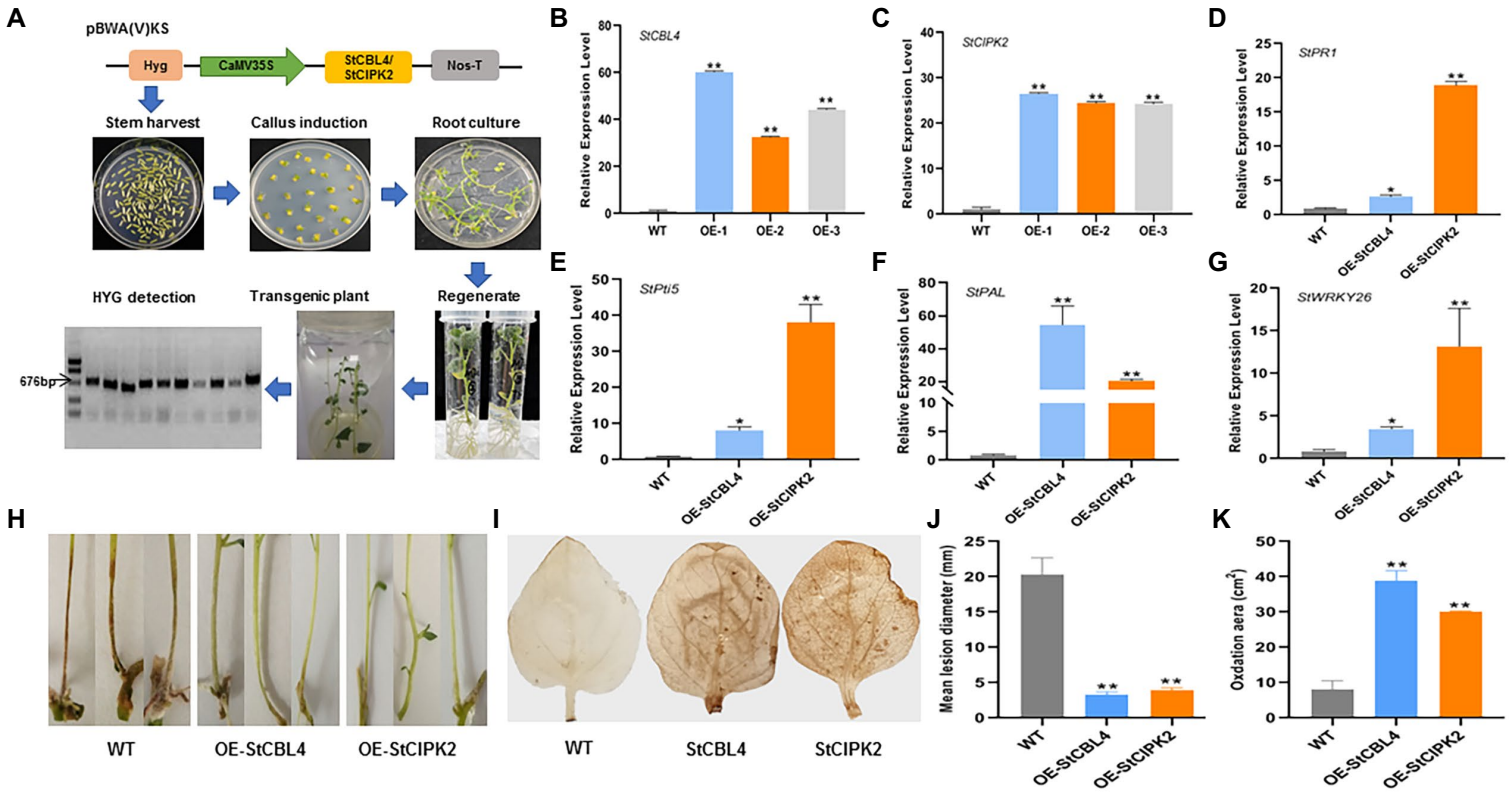
Generation and function analysis of Transgenic Plants on resistance to *R. solani*. **(A)** Generation of Transgenic Plants. Skeleton of pBWA(V)KS vectors was used for overexpressing *StCBL4* or *StCIPK2* gene. The schematic diagram shows the process of inducing plant. Target HYG was detected for the overexpressed positive plant. **(B,C)** Overexpression profile of *StCBL4* and *StCIPK2* gene in transgenic plant (OE-1, OE-2, and OE-3) by real-time RT-PCR. The relative mRNA levels were calculated with respect to the expression level of the corresponding gene in the WT plant. **(D)** The expression levels of *StPR1* were higher in StCBL4- and StCIPK2-overexpressing plants than WT plants after *R. solani* infection. **(E)** The expression levels of *StPti5* were higher in StCBL4- and StCIPK2-overexpressing plants than WT plants after *R. solani* infection. **(F)** The expression levels of *StPAL* were higher in StCBL4- and StCIPK2-overexpressing plants than WT plants after *R. solani* infection. **(G)** The expression levels of *StWRKY26* were higher in StCBL4- and StCIPK2-overexpressing plants than WT plants after *R. solani* infection. **(H)** Symptoms were observed on stems inoculated with *R. solani*. **(I)** DAB staining of H_2_O_2_ accumulation in leaves of a transgenic plant. **(J)** Mean lesion diameter measured after *R. solani* inoculation at 10 days post-inoculation. **(K)** The amount of H_2_O_2_ produced was measured by calculating the DAB-stained area, in which the dark yellow flecks indicated the ROS burst sites. All the experiments were repeated three times, and similar results were obtained. The data represent the means ± SD of triplicate measurements (*n* > 10). Asterisks above the columns represent significance based on an unpaired, two-tailed Student’s *t*-test relative to the control. *p < 0.05; **p < 0.01.

Given that resistance to *R. solani* was enhanced in *N. benthamiana* that overexpressed *StCBL4* and *StCIPK2*, we next generated transgenic potato cultivar Désirée lines expressing *StCBL4* and *StCIPK2* to investigate the functions of these genes further. As shown process in Figure 6A, transgenic plants were obtained and confirmed *via* RT-PCR detection of *hygromycin* transcripts. Empty pBWA(V)KS vector-transformed plants served as wild-type (WT) controls.

The results of the qRT-PCR analysis revealed >30 and > 20-fold upregulation of *StCBL4* and *StCIPK2* expression in overexpressing plants, respectively (Figures 6B,C). Subsequently, WT and overexpressing transgenic plantlets were incubated with 5-mm-diameter mycelium plugs. Then the plants were grown under identical conditions, and were observed for symptom development after 3 dpi. Compared with the visible symptom of WT plantlets, fewer transgenic plantlets showed canker symptoms at 10 dpi (Figure 6H). As shown in Figure 6J, lesion diameters were measured and showed to be about 16 and 20% lower than corresponding levels in WT. These findings indicated that overexpression of these genes in transgenic plantlets could potentially delay canker development to alter the course of the *R. solani* infection.

Accumulation of ROS, a significant plant regulatory factor, is an important indicator of plant resistance, especially during the early stage of infection. ROS plays important roles in early signaling events initiated by cellular metabolic perturbation and environmental stimuli (Lehmann et al., 2015). To verify whether StCBL4 and StCIPK2 are involved in H_2_O_2_ signaling, ROS bursts were induced in both *StCBL4*- and *StCIPK2*-overexpressing plants then H_2_O_2_ production was analyzed by analyzing images of DAB-stained leaves. As shown in Figure 6I, a ROS burst was observed by the dark yellow flecks in each overexpressing plants. The amount of H_2_O_2_ produced was measured to be about 4.84- and 3.74-fold higher than the corresponding levels in WT by calculating the DAB-stained area, respectively. The results revealed that both StCBL4 and StCIPK2 participate in H_2_O_2_ signaling.

### Defense genes are upregulated in transgenic potato expressing *StCIPK2* and *StCBL4*

To understand molecular regulation underlying increased resistance to the *R. solani* pathogen, expression levels of plant defense genes were measured in infected *StCBL4*- and *StCIPK2*-overexpressing plants at 3 dpi. Defense genes that were studied included pathogenesis-related protein 1 (*StPR1*), Pto interaction protein 5 (*StPti5*), phenylalanine ammonia lyase (*StPAL*), pathogenesis-related protein 10 (*StPR10*), WRKY transcription factor 26 (*StWRKY26*), and their expression patterns were examined by qRT-PCR. The results revealed that expression levels of *StPR1* were upregulated by 2.67- and 18.9-fold in *StCBL4*- and *StCIPK2*-overexpressing plants compared with levels in WT plants. Meanwhile, expression levels of *StPti5*, *StWRKY26*, and *StPAL* were found to be 8.14- and 38.17-fold, 3.49- and 13.18-fold, and 54.46- and 20.14-fold higher in *StCBL4* and *StCIPK2*-overexpressing transgenic plants, respectively. Furthermore, compared with *StCBL4*-overexpressing plants, higher *PR* gene expression levels (except for that of *StPAL*) were observed in *StCIPK2*-overexpressing plants (Figures 6D–G).

## Discussion

*Rhizoctonia solani* causes stem canker and black scurf, one of the most severe soil-borne fungal diseases of potato that dramatically reduce yield and quality. Breeding for disease-resistant varieties is viewed as the most effective and eco-friendly method for disease control, with potato disease resistance shown in a previously reported study to rely on *R. solani* infection-induced triggering of plant pathways that induce production of hormones jasmonic acid (JA) and salicylic acid (SA; Zrenner et al., 2021). However, as no endogenous resistance gene against *Rhizoctonia* disease has yet been identified in potato, the molecular response of potato to *R. solani* attack remains largely unknown. Our present study found that *StCBL4* and *StCIPK2* positively enhance potato anti-fungal responses for combating *Rhizoctonia* disease.

Although the role of the CBL-CIPK complex in regulating the response against abiotic stress is well documented, it remains unclear whether this complex also regulates responses against biotic stress. In a previous study, several CBL–CIPK interaction partners were hypothesized (based on limited evidence) to play roles in biotic and/or oxidative stress responses and developmental pathways (Pandey et al., 2014). For example, the interaction between OsCIPK14/15 and OsCBL4 plays a crucial role in enhancing rice resistance to *Trichoderma viride* ethylene-inducing xylanase (Kurusu et al., 2010). The TaCBL4-TaCIPK5 complex acts to promote ROS signaling to enhance the resistance of wheat to *Puccinia triiformis* f. sp. *tritici* (Liu et al., 2018). Since CBL4 has been reported to participate in biotic stress resistance functionally, we investigated whether StCBL4 may interact with StCIPK2 to perform similar functions in potato. In this study, transient overexpression of *StCBL4* and *StCIPK2* individually and synergistically increased the tolerance of potato plants to *R. solani* in *N. benthamiana*, meanwhile transgenic potato significantly enhanced resistance as well (Figures 2 and 5B,C,H,J); thus implying that these two genes play crucial roles in defensive responses and innate immunity in potato.

To further confirm that *StCBL4* and *StCIPK2* genes mediate the interaction between plant and *R. solani*, TRV-VIGS was used to successfully knock down the expression of *StCBL4* and *StCIPK2* orthologs *NbCBL* and *NbCIPK2*, respectively, that are found in *N. benthamiana* (Supplementary Figure 3). Notably, silencing of these genes in detached leaves followed by inoculation of leaves with *R. solani* led to larger lesions in plants with knocked down *NbCBL* and *NbCIPK2* expression compared to unsilenced controls. This result indicates that the knockdown of *NbCBL* and *NbCIPK2* expression increased sensitivity to *R. solani* infection (Figures 3C,D). Importantly, similar results were observed in wheat, where knockdown of TaCBL4 and TaCIPK5 expression led to a reduced wheat defense response against stripe rust fungus (Liu et al., 2018).

The abovementioned results notwithstanding, the mechanism responsible for the effects of these two proteins on plant resistance remains unknown. However, StCIPK2, an interaction partner of StCBL4, was identified based on results obtained *via* Y2H assays that were subsequently confirmed *via* LCA and Co-IP assays (Figure 4). Furthermore, previously reported results have shown that combinations of CBLs and CIPKs functions act as signaling nodes in response to environmental stimuli (Thoday-Kennedy et al., 2015). However, the CIPK protein sequence lacks localization motifs, subcellular localization of CBL-CIPK modules depends on myristoylation and acylation modifications of CBL sites (Mao et al., 2022). In this study, StCBL4 was found to interact with StCIPK2 to specifically recruit StCIPK2 to the plasma membrane in *N. benthamiana* cells, thus indicating that StCIPK2 interacted with a potentially myristoylated StCBL4 site as a necessary and sufficient condition for StCIPK2 anchoring to the plasma membrane to occur.

Production and accumulation of defense-related proteins in plants in response to biotic stresses are well-known crucial plant defense mechanisms (Wang et al., 2022). Salicylic acid (SA), a key signaling plant resistance molecule involved in resistance to biotrophs, induces the expression of pathogenesis-related (PR) proteins that induce systemic acquired resistance (SAR). As compared with their expression in WT plants, expression levels of PR protein-encoding genes (e.g., *PR1*, *Pti5*, and *WRKY26*) were up-regulated significantly in both *StCBL4*- and *StCIPK2*-transgenic plants, thus indicating that *StCBL4* and *StCIPK2* participated in potato immune response to defend plants against the pathogen (Figures 6D,E,G). *PAL* is a critical gene in the phenylalanine metabolic pathway that effectively regulates the biosynthesis of corresponding secondary metabolites (Wang et al., 2021). In this study, high-level expression of *StPAL* revealed the role of an important downstream signaling node as a potential molecular mechanism underlying plant resistance to *R. solani* disease (Figure 6F).

The accumulation of reactive oxygen species (ROS) is a widespread defense mechanism in higher plants against pathogen attack and sometimes is the cause of cell death that facilitates attack by necrotrophic pathogens (Tian et al., 2021). As well known, necrotrophic fungi with a wide host range can kill host plant cells and use the contents to support their growth. However, despite their substantial role in crop losses, there is much less known about these pathogens and their ability to infect. As a necrotrophic fungus, *R. solani* could cause physical changes including stunted root growth of the host, transcriptional changes and ROS production (Foley et al., 2016). ROS has multiple functions in plant defense serving as a signaling molecule. It has been proved that H_2_O_2_, one of the important ROS productions, plays a role in plant defense as a messenger in signal transduction pathways linked to oxidative stress signaling (Huang et al., 2021). In our study, *StCBL4* and *StCIPK2* showed the highest induction in transgenic potato plants coinciding with the induction of H_2_O_2_ accumulation (Figures 6B,C,I,K). This observation agrees with previous studies that demonstrated that the function of many CBL-CIPK complexes in abiotic and biotic stress signaling is associated with the burst of ROS. Therefore, research focusing on the accumulation of ROS of fungal necrotrophs will also help to understand their pathogenic mechanisms on plants. Indeed, lots of previous research focusing on the RBOH family has confirmed the interaction with CIPKs (Sanyal et al., 2020). Thus, we also speculate that the ROS generation mediated by the StCBL4-StCIPK2 complex during potato resistance to *R. solani* potentially depends on RBOH proteins. Moreover, identifying which remember of RBOH family plays a role with StCIPK2 should be determined in further studies.

In conclusion, this studyhas illustrated that *StCBL4* and *StCIPK2* are involved in regulating the potato defensive immune response against *R. solani*. Furthermore, our results demonstrate that *StCBL4* functions in concert with *StCIPK2*, as positive regulators of immunity and contribute to combating stem canker disease in potato.

## Data availability statement

The datasets presented in this study can be found in online repositories. The names of the repository/repositories and accession number(s) can be found in the article/Supplementary material.

## Author contributions

SY and XW conceived and designed the experiments. SY, JLi, JLu, QW, WW, XD, and YM performed the experiments. SY, FM, MG, LH, and LW analyzed the data. SY, JLi, and XW wrote and revised the paper. All authors contributed to the article and approved the submitted version.

## Funding

This work was supported by the Natural Science Foundation of Heilongjiang Province (YQ2019C022) and Key-Area Research and Development Program of Guangdong Province (2020B020219002).

## Conflict of interest

The authors declare that the research was conducted in the absence of any commercial or financial relationships that could be construed as a potential conflict of interest.

## Publisher’s note

All claims expressed in this article are solely those of the authors and do not necessarily represent those of their affiliated organizations, or those of the publisher, the editors and the reviewers. Any product that may be evaluated in this article, or claim that may be made by its manufacturer, is not guaranteed or endorsed by the publisher.

## Footnotes

^1^ http://smart.embl-heidelberg.de/

^2^ https://imagej.nih.gov/ij

^3^ http://vigs.solgenomics.net/

## Supplementary material

SUPPLEMENTARY FIGURE 1 Amino acid sequence alignment and phylogenetic tree analysis of potato StCBL4 with other crops CBL4. **(A)** Amino acid sequence alignment. **(B)** Phylogenetic tree analysis. StCBL4(ON383960): *Solanum tuberosum* CBL4; AtCBL4(AF192886): *Arabidopsis thaliana* CBL4; CaCBL4(XM_016694246): *Capsicum annuum* CBL4; CsCBL4(XM_004146172): *Cucumis sativus* CBL4; OsCBL4(DQ201198): *Oryza sativa* CBL4; TaCBL4(KU736850): *Triticum aestivum* CBL4; ZmCBL4(EU973091): Zea mays CBL4; VvCBL4(KT266265): *Vitis vinifera* CBL4; HvCBL4(HM175879): *Hordeum vulgare* CBL4.

SUPPLEMENTARY FIGURE 2 Amino acid sequence alignment and phylogenetic tree analysis of potato StCIPK2 with other crops CIPK2. **(A)** Amino acid sequence alignment. **(B)** Phylogenetic tree analysis. StCIPK2 (ON324205): *Solanum tuberosum* CIPK2; AtCIPK2 (AF192886): *Arabidopsis thaliana* CIPK2; OsCIPK2 (EU703793): *Oryza sativa* CIPK2; TaCIPK2 (KU736853): *Triticum aestivum* CIPK2; HvCIPK2 (KP638475): *Hordeum vulgare* CIPK2; NtCIPK2 (XM_016647791): *Nicotiana tabacum* CIPK2.

SUPPLEMENTARY FIGURE 3 Phylogenetic relationship of CBLs and CIPKs from potato, *Arabidopsis* and tobacco. The Neighbor-joining (NJ) tree was constructed using MEGA 5.0 software. The black triangle and circle represent StCBL4 and StCIPK2 from potato, respectively. **(A)** Phylogenetic tree analysis of CBLs from potato, *Arabidopsis* and tobacco. **(B)** Phylogenetic tree analysis of CIPKs from potato, *Arabidopsis* and tobacco.

SUPPLEMENTARY FIGURE 4 Sequence alignment of StCBL4 with NbCBL and StCIPK2 with NbCIPK2. Sequence alignment was performed by DNAMAN 6.0 software. The red line represents the VIGS site searched by SGN VIGS Tool.

SUPPLEMENTARY TABLE 1 The list of various potato germplasm resources.

SUPPLEMENTARY TABLE 2 Primers used in this study.
