# Supplementary figures and images for: Potato calcineurin B-like protein CBL4, interacting with calcineurin B-like protein-interacting protein kinase CIPK2, positively regulates plant resistance to stem canker caused by Rhizoctonia solani
Source: Front Microbiol. 2023 Jan 4;13:1032900. doi: 10.3389/fmicb.2022.1032900 (PMC9845770; doi:10.3389/fmicb.2022.1032900)

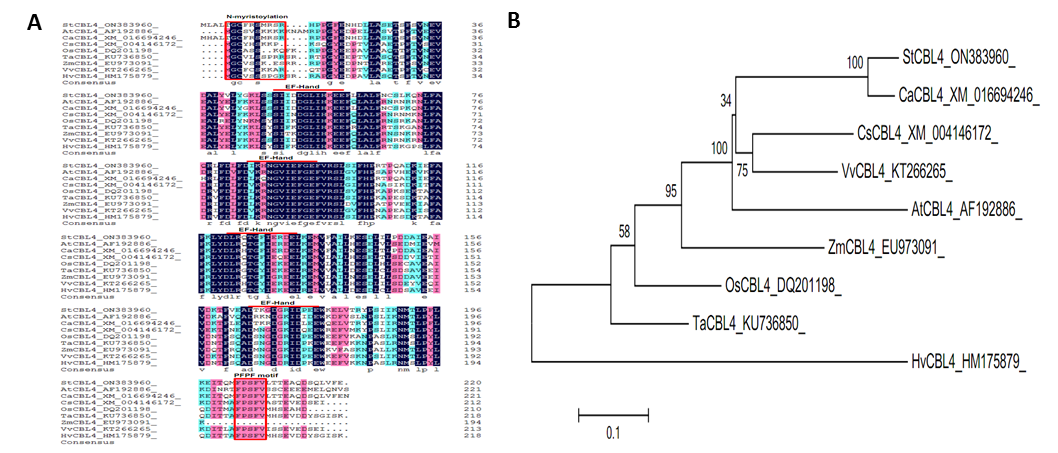

Supplement: SUPPLEMENTARY FIGURE 1 — Amino acid sequence alignment and phylogenetic tree analysis of potato StCBL4 with other crops CBL4. (A) Amino acid sequence alignment. (B) Phylogenetic tree analysis. StCBL4(ON383960): Solanum tuberosum CBL4; AtCBL4(AF192886): Arabidopsis thaliana CBL4; CaCBL4(XM_016694246): Capsicum annuum CBL4; CsCBL4(XM_004146172): Cucumis sativus CBL4; OsCBL4(DQ201198): Oryza sativa CBL4; TaCBL4(KU736850): Triticum aestivum CBL4; ZmCBL4(EU973091): Zea mays CBL4; VvCBL4(KT266265): Vitis vinifera CBL4; HvCBL4(HM175879): Hordeum vulgare CBL4. [file Image_1.TIF]

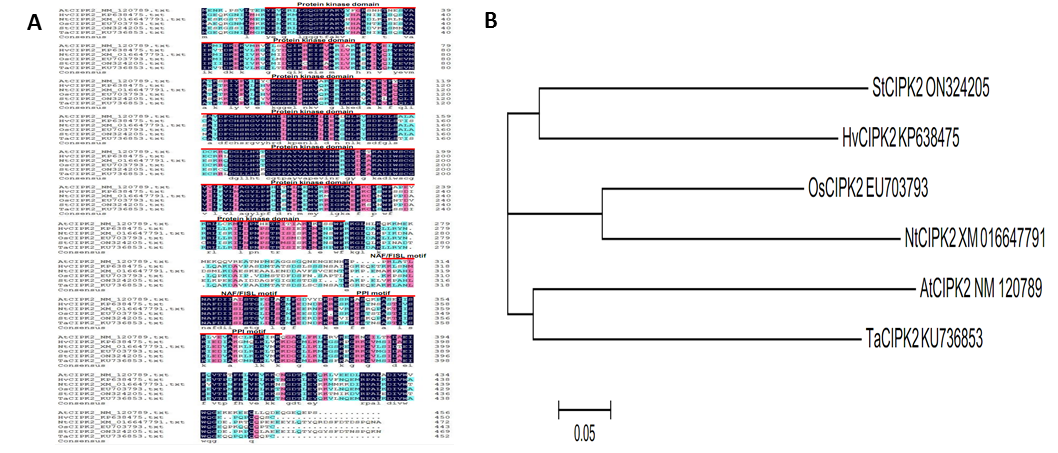

Supplement: SUPPLEMENTARY FIGURE 2 — Amino acid sequence alignment and phylogenetic tree analysis of potato StCIPK2 with other crops CIPK2. (A) Amino acid sequence alignment. (B) Phylogenetic tree analysis. StCIPK2 (ON324205): Solanum tuberosum CIPK2; AtCIPK2 (AF192886): Arabidopsis thaliana CIPK2; OsCIPK2 (EU703793): Oryza sativa CIPK2; TaCIPK2 (KU736853): Triticum aestivum CIPK2; HvCIPK2 (KP638475): Hordeum vulgare CIPK2; NtCIPK2 (XM_016647791): Nicotiana tabacum CIPK2. [file Image_2.TIF]

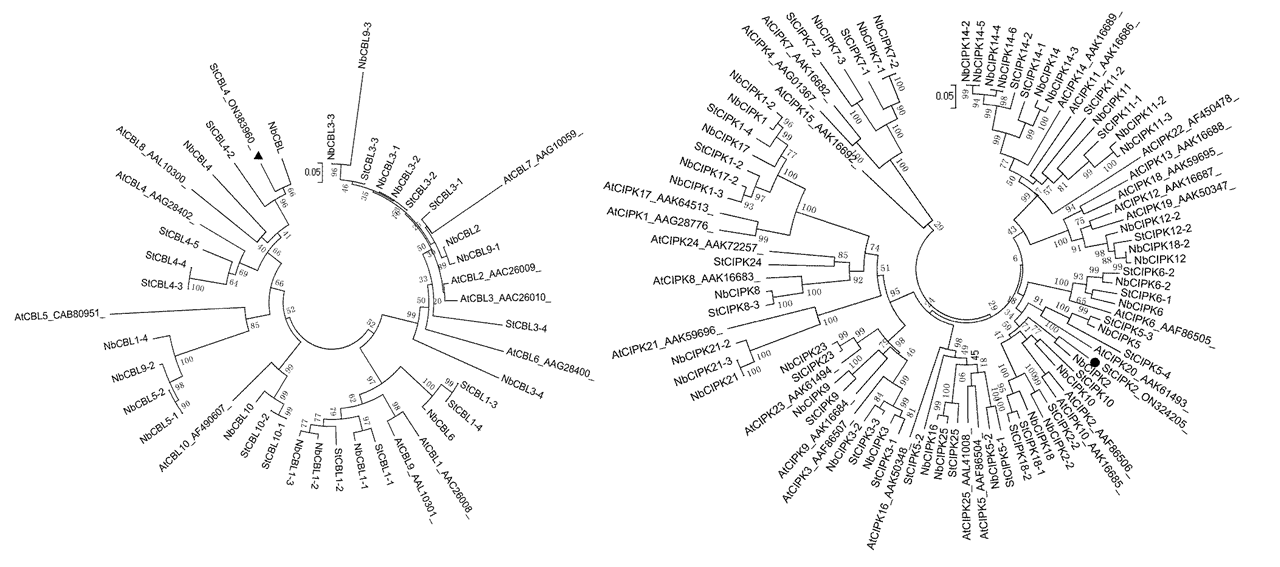

Supplement: SUPPLEMENTARY FIGURE 3 — Phylogenetic relationship of CBLs and CIPKs from potato, Arabidopsis and tobacco. The Neighbor-joining (NJ) tree was constructed using MEGA 5.0 software. The black triangle and circle represent StCBL4 and StCIPK2 from potato, respectively. (A) Phylogenetic tree analysis of CBLs from potato, Arabidopsis and tobacco. (B) Phylogenetic tree analysis of CIPKs from potato, Arabidopsis and tobacco. [file Image_3.TIF]

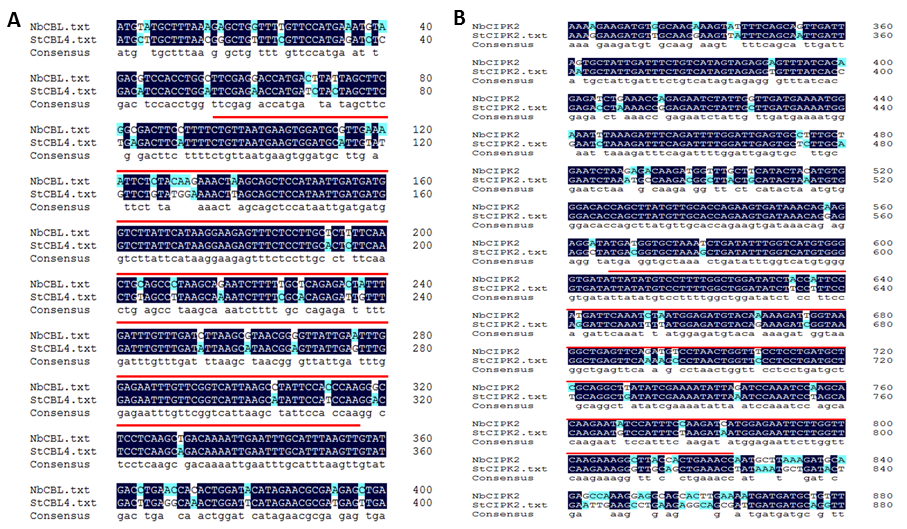

Supplement: SUPPLEMENTARY FIGURE 4 — Sequence alignment of StCBL4 with NbCBL and StCIPK2 with NbCIPK2. Sequence alignment was performed by DNAMAN 6.0 software. The red line represents the VIGS site searched by SGN VIGS Tool. [file Image_4.TIF]
